# How Culture Enables Practice: Facilitators and Barriers to the Use of Total Intravenous Anesthesia in Pediatric Anesthesia

**DOI:** 10.1002/pan.70214

**Published:** 2026-05-11

**Authors:** Nicholas West, Eleanor Reimer, Asli E. Ozer, Rotem Killam, Matthias Görges

**Affiliations:** ^1^ Department of Anesthesia BC Children's Hospital Vancouver British Columbia Canada; ^2^ Research Institute BC Children's Hospital Vancouver British Columbia Canada; ^3^ Department of Anesthesiology, Pharmacology & Therapeutics The University of British Columbia Vancouver British Columbia Canada

**Keywords:** change management, education, healthcare systems, implementation science, intravenous anesthesia, pediatric anesthesia

## Abstract

**Introduction:**

Total intravenous anesthesia (TIVA) in children has gained popularity due to potential advantages, including decreased respiratory adverse events, postoperative nausea and vomiting, and emergence agitation. British Columbia Children's Hospital (BCCH) has a reputation for TIVA use, training, and advocacy, including intravenous induction.

**Aim:**

To inform practice change and education by exploring how institutional culture impacts TIVA adoption, particularly regarding intravenous cannulation in awake children.

**Methods:**

Current and former BCCH anesthesiologists and trainees were surveyed and interviewed about TIVA practices at BCCH and at their current institutions. The survey covered demographics, practice settings, past and current methods for induction and maintenance of anesthesia, factors contributing to technique selection, TIVA barriers and facilitators, and implementation factors. Semi‐structured interviews conducted with a subset of participants were analyzed using deductive and inductive thematic analysis.

**Results:**

Twenty‐six participants completed the survey: 21 attending physicians, 2 fellows, and 3 residents; 58% with < 10 years' and 23% with > 20 years' anesthesia practice. Most participants (92%) practised in an academic setting, caring for a median [interquartile range] 400 [200–500] children annually. Most (96%) indicated their BCCH experience had changed their practice, with a greater effect on using intravenous anesthesia for maintenance than induction. Changes were influenced by a positive experience (77%), supportive environment (42%), and scientific evidence (42%). Choice of anesthetic technique depended on patient factors (89%), institutional expectations (46%), pharmacology (42%), and patient preference (39%), but not parental preference (15%). Interviews with 11 participants focused on intervention bundles to enable success, expectation‐setting, family education, equipment and staff availability, and supportive workflows.

**Conclusion:**

Training in a TIVA institution can have a profound effect on using TIVA in children and drive changes in anesthetic practice elsewhere. Introducing TIVA requires a supportive culture, appropriate equipment, and the personalization of approaches, and may benefit from understanding factors in change management.

## 1 Introduction

Total intravenous anesthesia (TIVA) has gained popularity among pediatric anesthesiologists [1], at least partly due to its potential advantages over inhalational anesthesia [2]. Strictly, TIVA requires intravenous (IV) cannulation in an awake child to facilitate both an IV induction and IV maintenance of anesthesia. However, a common alternative consists of an inhalational induction followed by IV maintenance; this is sometimes considered sufficient to apply the label “TIVA” [3], but may be more usefully characterized as mostly IV anesthesia (MIVA).

While inhalational induction with oral sedative premedication remains the most commonly used mode of induction for pediatric anesthesia in North America and most of Europe [4, 5], IV induction is used frequently at BC Children's Hospital (BCCH), with 57% of patients having TIVA and a further 20% having MIVA [6]. BCCH has gained an international reputation for training, using, and advocating for this approach.

In a 2019 study, interviews with anesthesiologists and procedural suite nurses identified success factors for IV induction of anesthesia in children, including effective distraction, family preparation, parental presence, support of the operating room team, effective use of local analgesic cream, adapting the approach to the individual child, and the anesthesiologist's efficiency in cannulation; barriers included needle phobia, uncooperative children, anxious parents, ineffective use of analgesic cream, and unfavorable anatomy [6].

This study aims to learn how institutional culture affects the use of TIVA in children, particularly regarding the inclusion of IV induction of anesthesia within this approach, to inform future implementation and expansion and to guide future teaching. Exploring the superiority (or otherwise) of TIVA in general or specific cases is outside the scope of this study. Rather, we aim to learn (a) if and how experience at our institution changes practitioners' subsequent approaches, (b) to what degree this experience contributes to changing approaches to TIVA, and (c) if and how a different local institutional culture enables or limits the adoption of new TIVA practices.

These inquiries frequently focus on awake cannulation in children prior to IV induction of anesthesia, as this is the activity in which TIVA intersects most clearly with colleagues' practice and institutional culture.

## 2 Methods

### 2.1 Study Design and Approval

We conducted a 2‐phase study: (1) a survey of current and former anesthesiologists and advanced anesthesia trainees at BCCH; (2) interviews with a representative sample from the same population. The study was approved by the University of British Columbia (UBC) Children's and Women's Health Centre of BC Research Ethics Board (H23‐03017, approved 2023‐12‐07). Data were collected between December 2023 and September 2024. This manuscript was guided by the Consensus‐based Checklist for Reporting of Survey Studies (CROSS) [7] and Consolidated Criteria for Reporting Qualitative Research (COREQ) [8].

### 2.2 Participants

The survey was distributed to participants, who were required to self‐identify as belonging to one of two groups: (a) anesthesiologists and fellow physicians currently working at BCCH; (b) anesthesiologists and anesthesia trainees (senior residents or fellows) who had trained or worked at BCCH. Author MG contacted all attending anesthesiologists, the four current anesthesia fellows and all fellows who had trained in the BCCH Department of Anesthesia since 2010 via email; they also shared the survey with physicians who had retired or left the department since 2010. The UBC anesthesia residency program director distributed the survey to all senior residents and those who had graduated in the past 2 years.

The survey informed potential participants about the study, including its risks and benefits; consent was implied by completing the survey. No remuneration was offered to survey participants. Participants who were willing to be contacted for a follow‐up interview provided consent via the Research Electronic Data Capture (REDCap) electronic consent framework [9, 10]; consent was affirmed at the start of the interview. Interview participants were remunerated with a $25 CAD gift card.

### 2.3 Data Collection

#### 2.3.1 Survey

The survey was delivered via a public REDCap survey link, so responses were submitted anonymously unless the respondent provided their email address for a follow‐up interview or included identifying information in their free‐text responses. The questionnaire included basic demographic questions, level of training, and current practice, followed by questions on perceived enablers and barriers to using TIVA, and open‐ended questions (Appendix S1, survey).

#### 2.3.2 Interview

Semi‐structured interviews were conducted remotely via Zoom's online videoconferencing service (Zoom Communications Inc., San Jose, CA). The interview followed a structured guide, exploring how the effects of working and training at BCCH had changed or were changing their perioperative practice, institutional culture, and some of the barriers and facilitators to potential TIVA program implementation (Appendix S2, interview guide). Interviews were recorded and lasted 30 min–1 h.

Interviews were conducted by two investigators, a former BCCH staff anesthesiologist (female, 30 years of experience in anesthesia and research; existing relationship as former department head and/or colleague with all participants; author ER) and a research coordinator (male; 13 years of experience in clinical research, existing relationship as research assistant to some participants; author NW).

### 2.4 Data Analysis

For the survey, we aimed to include a convenience sample of 50 participants, including 20 anesthesiologists and four fellow physicians currently practising at BCCH, 10 residents who trained at BCCH and graduated, and 15 fellows who had trained at BCCH. We then aimed to conduct 13–15 interviews with participants from the same populations.

Quantitative data collected through the survey were summarized and analyzed in R (R Foundation for Statistical Computing, Vienna, Austria) and reported as median with interquartile range (IQR) for continuous measures and as counts with percentages for categorical measures.

For qualitative responses from the interviews, we applied a grounded theory analysis approach [11], in which we identified key themes, patterns, and relationships in the data through iterative coding and analysis. Interview recordings were automatically transcribed (using Zoom's built‐in functions), transcriptions were checked by the interviewer to remove identifying details, and imported into NVivo 14 (Lumivero, Denver, CO). Data analysis was led by AO, who is a qualitative analyst, and supported by the co‐investigators. We performed an inductive‐deductive thematic approach [12], Analysis included an iterative process of data familiarization through reviewing transcripts, code generation through identification of data‐driven themes, theme searching and review by identifying groupings and overarching themes, theme definition by refining and determining the essence of each theme and finalization by exploring and describing the relationship among themes and subthemes [13].

## 3 Results

### 3.1 Survey: Participants

The survey invitation was sent to 32 anesthesiologists at BCCH, 13 former staff, 30 current and former fellows, and 47 current and 21 former residents (a total of 143 invited). Thirty‐one participants accessed the survey, and 26 completed all sections (response rate 18%), including 21 staff, 2 fellows, and 3 residents: 13 (50%) were male, 12 (46%) were under 40 years of age; 15 (58%) had been in practice for less than 10 years of experience and 6 (23%) had been in practice for over 20 years (Table 1). Most participants (24, 92%) now practised in an academic setting, caring for a median [IQR] of 400 [200–500] children annually.

**TABLE 1:** Survey respondent characteristics.

	Overall *N* = 26	Staff (current/retired) *N* = 21	Trainee (fellow/resident) *N* = 5
Gender: Male, *n* (%)	13 (50%)	10 (48%)	3 (60%)
Age, *n* (%)			
≤ 30 years	1 (4%)	0	1 (20%)
31–40 years	11 (42%)	8 (38%)	3 (60%)
41–50 years	7 (27%)	7 (33%)	0
51–60 years	4 (15%)	3 (14%)	1 (20%)
61–70 years	1 (0.4%)	1 (0.5%)	0
> 70 years	2 (8%)	2 (10%)	0
Years in practice, *n* (%)			
0–5 years	8 (31%)	6 (29%)	2 (40%)
5–10 years	7 (27%)	6 (29%)	1 (20%)
10–15 years	3 (12%)	2 (10%)	1 (20%)
15–20 years	2 (8%)	2 (10%)	0
> 20 years	6 (23%)	5 (24%)	1 (20%)
Practice setting: Community, *n* (%)	2 (8%)	2 (10%)	0
Number of pediatric cases per year, median [IQR]	400 [200, 500]	400 [200, 500]	300 [50, 400]
Ethnicity, *n* (%)			
Chinese	2 (8%)	1 (5%)	1 (20%)
Latin American	1 (4%)	1 (5%)	0
South Asian	2 (8%)	2 (10%)	0
White	20 (77%)	17 (81%)	3 (60%)
Other	1 (4%)	1 (5%)	0
Prefer not to answer	1 (4%)	0	1 (20%)

### 3.2 Survey: Practice Change

**FIGURE 1:**
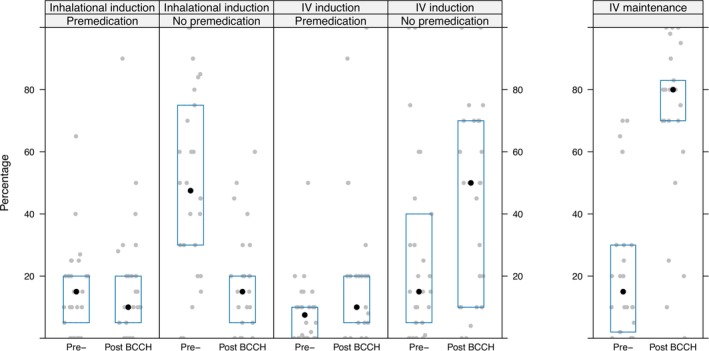
Reported changes in pediatric anesthesia practice patterns among survey responses comparing percentage of cases with each technique before practicing at BC Children's Hospital (Pre‐) versus after (Post BCCH). Data are displayed as boxplots showing median (black dot) with interquartile range (box) with individual percentages overlaid as gray dots.

Most participants (25, 96%) indicated that their BCCH experience had changed their practice (Table 2). The greatest effect was using TIVA for maintenance, which increased from 15 [3–30]% to 80 [70–82]%, though IV induction also increased from 30 [6–50]% to 70 [50–80]% (Figure 1). These changes were predominantly influenced by a positive experience at BCCH (20/26, 77%), a supportive environment (11, 42%), scientific evidence (11, 42%), and mentorship (10, 39%). Key factors influencing the choice of anesthesia technique included patient factors (23, 89%), current institutional expectations (12, 46%), pharmacology (11, 42%), and patient preference (10, 39%), but parental preference was least influential (4, 15%).

**TABLE 2:** Reasons to change practice and factors determining use of total intravenous anesthesia techniques among staff (current/retired) and trainee (fellow/resident) survey respondents.

	Staff (*N* = 21)	Trainee (*N* = 5)
Most compelling reasons for changing practice, *n* (%)		
Scientific evidence	9 (43%)	2 (40%)
Mentorship	8 (38%)	2 (40%)
Supportive learning environment	9 (43%)	2 (40%)
Departmental expectations	4 (19%)	2 (40%)
Positive experiences with this technique	17 (81%)	3 (60%)
Other: *desire to learn*, *increased familiarity*, *equipment availability*	2 (10%)	1 (20%)
Most significant factors influencing choice of pediatric anesthetic technique, *n* (%)		
Patient preference	8 (38%)	2 (40%)
Parental preference	4 (19%)	0
Patient factors, e.g., anticipated difficult airway, difficult IV access, etc.	19 (90%)	4 (80%)
Institutional culture or expectations	9 (43%)	3 (60%)
Availability of equipment, space, etc.	4 (19%)	3 (60%)
Pharmacology of anesthetic drugs	9 (43%)	2 (40%)
Other: *availability of trained staff*	1 (5%)	0
Critical success factors for awake cannula placement, *n* (%)		
Effective distraction	17 (81%)	5 (100%)
Preparing the family for IV induction	17 (81%)	4 (80%)
Parental presence	8 (38%)	3 (60%)
Support from the operating room team	17 (81%)	4 (80%)
Effective use of local analgesic cream	18 (86%)	4 (80%)
Adapting the approach to the individual child	16 (76%)	4 (80%)
The anesthesiologist's efficiency/speed of placing the IV	14 (67%)	4 (80%)
Other: *performing IV insertion in the pre‐op area*	1 (5%)	0
Main barriers to awake cannula placement, *n* (%)		
Needle phobia	20 (95%)	4 (80%)
Uncooperative child	17 (81%)	5 (100%)
Anxious parents	16 (76%)	3 (60%)
Ineffective use of analgesic cream	14 (67%)	3 (60%)
Unfavorable anatomy	14 (67%)	3 (60%)
Lack of support by the operating room team	7 (33%)	3 (60%)
Lack of support by the pre‐operative team	5 (24%)	1 (20%)
Lack of parental presence at the induction location	1 (5%)	0
Lack of time/resources for family education and preparation	6 (29%)	1 (20%)
Other: institutional practice, patient/parent expectation of inhalational induction, children who had previous bad experience with multiple IV attempts	3 (14%)	0

### 3.3 Survey: Success Factors and Barriers

Key factors for TIVA success included effective distraction techniques (85%), the use of local anesthetic cream (85%), family preparation (81%), and a supportive team (81%) (Table 2). Key barriers to IV induction of anesthesia were needle phobia (92%), an uncooperative child (85%), ineffective local anesthetic cream (65%), and anatomical features (65%). Free‐text comments focused on bundles of interventions to enable success, expectation‐setting and family education, availability of resources (equipment and staff), and supportive workflows. Participants also highlighted role modeling, practice in awake children, and appropriate patient selection.

### 3.4 Semi‐Structured Interviews

Thirteen survey respondents expressed an interest in participating in a semi‐structured interview. A further three former clinical fellows were contacted, based on expected interest. We interviewed 11 of these 15 potential participants between 2024‐04‐09 and 2024‐09‐10: 4 current BCCH staff, 5 former fellows now practising as staff elsewhere, 1 current fellow, and 1 retired anesthesiologist.

**FIGURE 2:**
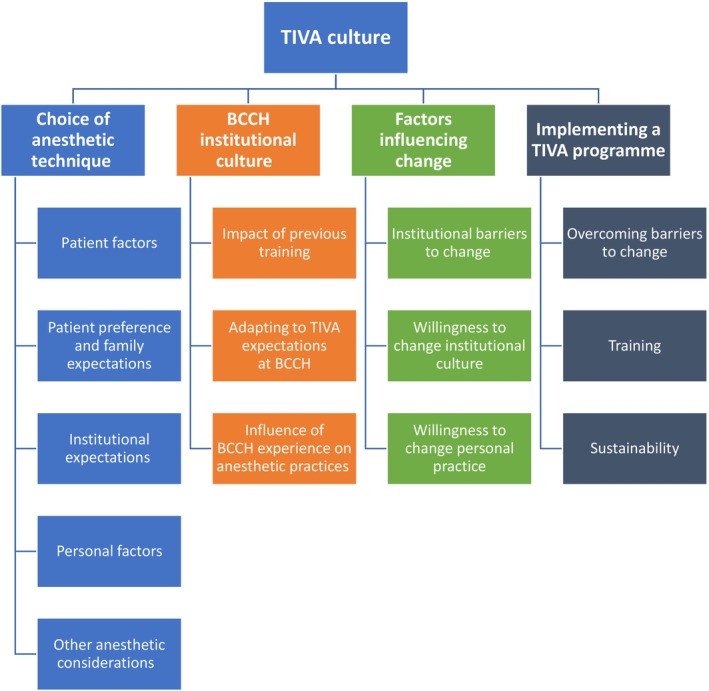
Themes and sub‐themes from thematic analysis of interviews.

Four main themes were constructed: (1) choice of anesthetic technique; (2) BCCH institutional culture; (3) factors influencing change, including the availability of resources (drugs and equipment); (4) implementing a TIVA program (Figure 2).

#### 3.4.1 Choice of Anesthetic Technique

This theme encompassed narratives related to participants' decision‐making processes and the factors influencing their choice of anesthetic technique. Participants identified patient factors as the primary consideration in selecting the anesthetic technique:

It's a function of the patient's medical history, relevant comorbidities, age and developmental stage. What kind of surgery they're having, what their risk factors for various potential complications are…

Participants also stressed the importance of considering patient and family preferences and expectations in choosing the method for induction of anesthesia:

If there's a strong family predilection, even a relatively minor one, for one way or the other, I'm usually happy to go that way. It is important to give families and children some autonomy and some ability to have some input into their care…

However, institutional expectations may also influence the selection of anesthetic technique:

… the existing culture and expectations, and maybe to some extent sort of mentorship and support from department members who are already doing this routinely […] are important influences.

Furthermore, participants noted that personal preferences and experience can also influence their decision‐making:

The more you do something a certain way or certain 2 or 3 ways, the more comfortable you get with the nuances of that technique, the better you get with refining that technique, and so […] most of us end up heading towards a small stable of techniques that we feel most comfortable with…

Finally, other anesthetic and surgical considerations were noted, such as using TIVA for cases requiring neurophysiological monitoring, auditory testing, or precise heart rate control:

Surgical factors. So, say I'm doing a case that requires neuro monitoring, then you would opt to use a TIVA‐based anesthetic. Or maybe you're doing a cochlear implant and they want to do some auditory testing at the end, or, you're doing a case that might really benefit from not having a ton of narcotic, but really might have good benefit from heart rate control, so you'd want to use some remifentanil, and I probably would choose TIVA in those cases as well.

#### 3.4.2 BCCH Institutional Culture

This theme encompassed narratives about the current institutional culture at BCCH, how participants adapted to it, and the ways BCCH has influenced their anesthetic practices. The impact of institutional expectations and culture was widely recognized:

Some things that BC Children's gets incredibly well are, you know, how the [anesthetic care unit] nurses will automatically put [local anesthetic cream] on, and you know they're very on board with trying to set us up for success with IV inductions, which is very different from anywhere else.

Participants noted the discrepancy between their previous experience and how anesthesia was predominantly practised at BCCH:

When I first came, I came from a training and a practice at […] and that was a majority inhalation induction practice, a majority inhalation maintenance practice, and the only people that we started awake IVs on were basically teenagers, ages 15 and up, so you might get one of those cases a couple times a month. Otherwise, everyone else got an inhalation induction and so, coming here and observing the local awake IV start practice and the local PR 5 infusion practice, my initial knee‐jerk reaction was, these people are crazy, and so I practise my old way for a while.

However, participants also described their experience of adapting to the expectations and culture within the BCCH Anesthesia department:

I felt supported. At first, it was scary, of course, but then I felt having everyone in the room used to doing it made me more comfortable with it as well, and then, after a few months, I would say I was comfortable getting IVs awake and knowing how to position the parent, how to distract the patient or knowing what patient would still need a pre‐med with that I got more comfortable…

Finally, participants recognized the influence of their experience at BCCH on their subsequent approach to pediatric anesthesia in other settings:

When I came back from BC Children's… I sort of recognize the value of TIVA, and I recognize that the way that BC Children's did it for the most part […] was IV induction and then TIVA, and I appreciated that, and I think there was value in that. And when I came back to the UK, I thought, yeah, this is what I'm going to do…

#### 3.4.3 Factors Influencing Change

This theme captured narratives about how institutional culture enables or hinders TIVA, participants' willingness to change, and the availability of resources, as well as non‐institutional factors like scientific evidence and environmental benefits. Participants acknowledged institutional barriers to change, including process challenges, such as patient expectation setting by nurses and surgeons and nursing experience with awake IV cannulation:

You have to set expectations with them, and if you tell them that they're going to have a gas induction and that they don't have to have an IV, then the ship has sailed, and it's very, very hard to then come in 5 min before they're due to go into the OR and change that expectation.

And then the nurses might not pick the best place for the [local anesthetic cream] because they're not so used to doing it, and then you're frustrated afterwards.

Another issue was the availability of necessary resources, including both drugs and equipment. For example, one interviewee noted that remifentanil had not been available in India until recently and even now was not always available due to local licensing issues. Other interviewees commented on the adoption of TIVA being constrained by the availability of infusion pumps and the challenges associated with obtaining this equipment:

Anything […] requires a massive fight to get any bit of equipment. So, we really had to put in […] business cases for getting pumps. So, when I first started there, we didn't have that many pumps and people were […] fighting over them.

The barrier to change personal practice was noted (“It's easier to turn on a volatile. It's easier to turn on a vaporizer than it is to prepare your TIVA”) and willingness to initiate change of institutional culture was varied. Some also recognized the value of practice consistency within a department:

Even though we work in silos in the operating room, we still work together as a team. So […] it is, I believe, good practice to have a solid group of people doing similar things in a department. I think that creates a bit of a level of safety, so that when someone has a problem in a room, they're not doing something so different that when three of their colleagues come in to help them, you know, it's out of keeping with their practice.

Several participants mentioned the need for compelling scientific evidence to change personal practices:

We all like to believe that we're providing evidence‐based care, and that the scientific literature supports whatever approach or approaches we choose […] the literature still talks about post‐op nausea and vomiting being the most common complication of inhalational anesthesia. We know that our PONV rates are less than 2%.

#### 3.4.4 Implementing a TIVA Program

This theme included narratives focused on overcoming barriers to implementing TIVA programs at various institutions, providing insights to guide future implementation strategies and education. Participants offered several suggestions for addressing challenges and initiating TIVA programs, with most emphasizing the importance of a whole‐team approach:

You have to […] get that buy‐in either from department heads or leaders in the department early on […] The second thing that you need is the buy‐in of the nursing staff and the child life staff. The entire system has to be introduced, and on board. The TIVA culture cannot succeed without everyone, all the way through the system, being familiar with and aware of the pros and cons of this technique.

Participants suggested demonstrating the feasibility and benefit of this approach in a sub‐population of patients:

[…] maybe institute a pilot [in a] subpopulation where you do that TIVA technique, and you get a subset of nurses and child life and surgeons and anesthetists on board with the technique and getting them to do it and then demonstrating the superiority in terms of outcomes and patient satisfaction.

Key training elements were identified, focusing on practical aspects of applying the technique and securing support from clinicians and caregivers, alongside the important consideration of ensuring program sustainability:

You have to teach them how to do a successful IV start on a younger kid, how to allay any anxiety around the needle insertion, how to distract well, how to prepare them with anxiolytics, if necessary, and how to get the parents involved in the distraction and in the positioning for successful IV start, and also selection of the patients, which patients don't you want to do an IV start on, so, understanding all that can take an entire rotation to do well.

We should […] teach it to the residents more formally, and part of the benefit of that is that then you can package those lectures and help other places induce this culture.

## 4 Discussion

This study, involving current and former staff and trainees, suggested that working in a department with an established reputation for TIVA had a significant impact on their subsequent use of TIVA in other institutions. This was mostly driven by a positive experience, existing scientific literature, availability of equipment, and a supportive culture. However, some anesthesiologists adopted a more limited use of TIVA, where it was not the established norm. Changing practice requires personal and institutional willingness to change, which depends on a range of factors, including obtaining the necessary equipment, demonstrating effectiveness, achieving buy‐in from anesthesia and other colleagues, and a constructive training approach (Table 3).

**TABLE 3:** Recommendations for implementing a Total Intravenous Anesthesia (TIVA) program.

#	Recommendation	Explanation and examples
1	**Equipment** Procure the necessary resources	Create a business case for procurement of infusion pumps and other equipment, citing evidence of benefit [2, 14]
2	**Support** Obtain buy‐in from anesthesia and other perioperative colleagues	First, seek support from department heads or leaders in the anesthesia department. Then find a willing subset of nurses, Child Life, and other allied health support staff, as well as surgeons and anesthesiologists. Familiarize them with the technique and anticipated benefits.
3	**Education** Adopt a constructive training approach	Develop a specific TIVA training program for residents and other staff. Focus on practical aspects and consider two major components: creating a practice comfortable with distress‐free awake IV starts, including how to: select appropriate patients; allay anxiety about needle insertion, prepare with anxiolytics, if needed; distract and get parents involved in distraction and positioning. training people on how to deliver an effective TIVA dosing regimen Consider (a) first then (b) or vice versa, rather than both together
4	**Pilot** Demonstrate effectiveness to the perioperative teams	Select a specific low‐risk pilot subpopulation in which you can demonstrate benefit, e.g., gastrointestinal endoscopy, and then demonstrate acceptability and perceived effect on outcomes and patient satisfaction
5	**Audit** Conduct a quality improvement (QI) program	After training has been established and local perioperative teams engaged, consider conducting a QI audit to investigate benefit to patients, families, and staff with local data

Despite evidence of benefit [2], research continues to examine why TIVA is not used more frequently [1, 15, 16]. A UK survey of pediatric anesthesiologists suggested that the main reason is that TIVA is too “fiddly”, followed by a lack of confidence, knowledge, or training, as well as concerns about awareness and the absence of target‐controlled infusion pumps [1]. An international survey similarly reported that “additional effort” was a significant concern; however, other barriers included lack of real‐time propofol concentration monitoring, difficulties with IV access, and ‘institutional preference’ [15]. A recent UK study found that, beyond clinical factors, the key drivers influencing anesthetic technique selection included time and efficiency pressures, patient experience, institutional defaults, and the influence of key decision‐makers [16].

Similarly, our participants identified the patient's physiology, anatomy, and planned surgery as key considerations in selecting their anesthetic technique, but recognized the importance of patient and family preferences and expectations, and acknowledged that institutional expectations, their own personal preferences, and experience also influenced their decision‐making. This is a complex mix. The pros and cons of adopting TIVA in a particular case in a particular setting cannot be resolved solely by appeal to scientific evidence [14, 17].

In most clinical settings, trainees must adapt to local expectations and culture while applying their previous understanding and experience. For our participants, the predominant anesthesia practices at BCCH typically differed from their previous experience, and the trainees had to adapt. While their BCCH experience influenced their subsequent approach, transferring a positive TIVA experience to their work elsewhere was not straightforward. The traditional narrative of anesthesia as a siloed field of individual independent decision‐makers does not apply to practices that necessarily involve other clinicians, resources, and the patient's perspective. Trainees may need to develop skills in change management [18].

Change barriers include the known problems of integrating new practices into existing processes (e.g., our participants identified expectation‐setting by nurses and surgeons, nursing experience with awake IV cannulation, and the availability of necessary equipment), as well as resistance from senior clinicians [19, 20]. Clinicians are typically unwilling to change their approach without compelling scientific evidence; they often value individual autonomy over group cohesiveness [21] and are unwilling to impose change on others [20]. While our participants recognized the value of patient experience and the safety of maintaining established routines, rigid conformity to existing practices can perpetuate resistance to change despite evidence‐based recommendations [22]. These finding are consistent with prior research on barriers and enablers of TIVA adoption [15, 16].

When considering how to initiate a TIVA program, our participants stressed the need to involve the entire clinical team, not just anesthesia. Effective change management often depends on both top‐down and bottom‐up strategies [20, 21, 23]. As noted, convincing senior colleagues, including department heads, may be challenging, but preliminary adoption of TIVA in a subpopulation of patients (e.g., patients undergoing gastrointestinal endoscopies) may provide an opportunity to gather local evidence of feasibility and effectiveness.

Education is essential to successful change management [19]. Key training elements should focus on the practical aspects of TIVA and on gaining the required support from other clinicians and caregivers, while also providing an understanding of the evidence and expected impact to achieve buy‐in [19, 24]. Guidance is available on implementation strategies to optimize uptake and sustainability [25]. Adoption of changes that make a task easier or more efficient may be intrinsically motivated, but otherwise implementation may need reinforcement with incentives and feedback [19, 20, 21], such as performance evaluations, audits, or reports providing measurable evidence of implementation success and its effect on outcomes [20, 22, 25].

## 5 Limitations

Our study has limitations. We report a small sample of surveys from a single institution, although the response rate was approximately a quarter of the invited participants. Our study explored the opinions of anesthesiologists during or after their experiences at a TIVA‐focused institution and lacked a substantial external reference, which could have been provided by comparing them with an institution with a different culture.

Our investigation focused on TIVA, and many of our findings addressed issues surrounding cannulation in awake children. This entailed the underlying assumptions that TIVA is, in many cases, a better approach to pediatric anesthesia and that IV induction is a necessary component of TIVA. While there is evidence for this viewpoint, it is not universally accepted [14], and some practitioners and guidelines may adopt a looser definition of TIVA, requiring only IV maintenance [3]. Our exploration of change management focused on IV cannulation and equipment issues, as these are the areas where TIVA intersects most clearly with institutional culture and resources. Some of our findings may be generalizable to other applications, but not all. While our findings offer some practical insights for TIVA users, they do not offer a comprehensive catalogue of methods or a roadmap for change.

Some of our survey questions used ranking language, such as “most significant” and “most compelling”, without clear selection limits, which may have prompted respondents to list only top priorities rather than their full set of considerations. There may have been a response bias among our interviewees: those more committed to TIVA may have been more willing to discuss the topic further, and our following up with former fellows based on expected interest may have reinforced this bias. One of the interviewers had previously supervised some of the interviewees, which may have influenced their responses.

## 6 Conclusion

Training in a TIVA institution can have a profound effect on the use of TIVA in children and can motivate clinicians to change their anesthetic practice in other institutions. However, introducing TIVA requires access to the appropriate equipment, a supportive culture among anesthesia and other clinical colleagues, and may require overcoming institutional barriers to change, especially regarding perioperative practices that facilitate IV induction of anesthesia. Further work is required to develop a set of educational and implementation resources to support this process.

## Funding

M.G. held a Michael Smith Health Research BC scholar award (SCH‐2020‐0494) during the conduct of this study and was supported by a 2020 BC Children's Hospital Research Institute External Salary Recognition Award.

## Ethics Statement

The study was approved by the UBC Children's and Women's Health Centre of BC Research Ethics Board (H23‐03017).

## Conflicts of Interest

The authors declare no conflicts of interest.

## Supporting information


**Appendix S1:** Survey consent and questions.

**Appendix S2:** Interview guide.

## Data Availability

The data that support the findings of this study are available on request from the corresponding author. The data are not publicly available due to privacy or ethical restrictions.
