# Supplementary material for: How Culture Enables Practice: Facilitators and Barriers to the Use of Total Intravenous Anesthesia in Pediatric Anesthesia
Source: Paediatr Anaesth. 2026 May 11;36(9):1078–88. doi: 10.1002/pan.70214 (PMC13460883; doi:10.1002/pan.70214)
Supplement: Supplementary file 1 [file PAN-36-1078-s001.pdf]

TIVA culture Appendix B – Interview guide

Note: Adjust questions and language of questions (wording, terminology, complexity) based on the respondent being interviewed.

Thank you for taking the time to sit down with us. We appreciate your participation in this study. My name is [*facilitator name*], and joining us is/are my fellow researcher(s) [*notetaker name(s) if present*]. We are team members working with Dr. Eleanor Reimer at BC Children's Hospital (BCCH) and the University of British Columbia.

Our study investigates how departmental and organizational culture enables total intravenous anesthesia (TIVA) in children to inform implementation strategies and guide future teaching. We aim to evaluate and learn more about some of the barriers and facilitators to potential TIVA program implementation, institutional culture change, and how the effect of working and training at BCCH has changed or is changing your personal perioperative practice.

The interview will last roughly forty-five to sixty minutes and will be recorded for further analysis. This interview is confidential, and your name and contact information will be kept separate from the other information you provide in this interview. Recordings and notes of your interview will be kept in a secure database, and only the research team will have access.

You can skip any questions you prefer not to answer and end the interview anytime. You can withdraw from the study even after completing the interview by asking us to remove your interview and information from the database. Do you have any questions about the interview?

Before we start, please confirm your consent to participate and be recorded.

[If consent is not confirmed, thank the participant for their time and end the session.]
[Proceed if consent is confirmed]

Introduction and background

Note to interviewer: *This set of questions is a guide only. It will not be possible to ask them all. Questions selected from each question will be guided by context, previous answers, and responses to the survey. Please ensure that at least one question from each section is asked.*

You completed an online survey before coming to this interview, and this interview will explore some of these topics in more detail. We only want you to answer questions you are comfortable with, and there is no right or wrong answer for some of the questions.

- Can you introduce yourself and briefly describe your role?

Willingness to change

- What are the most significant factors influencing your choice of anesthetic technique for pediatric patients?
- How did your experience at BCCH contribute to your changes in pediatric induction or maintenance practices?

Implementation

Assume you wanted to implement a TIVA program at your current or future institution.

- What would be the most significant barriers to success, and how would you suggest planning to overcome them?
- What are the critical components in their education, skills, and practices when bringing new learners or staff into your program to enable them to use TIVA in pediatric patients successfully?

Institutional Culture

- What aspects of institutional support would enable or hinder such a change?
- What are the most compelling arguments to change: patient/family preference, changes in scientific evidence, mentorship and supportive learning environments, departmental expectations, etc.?

Conclusion

- Are there any topics or concepts you missed or expected us to discuss?
- Do you have anything else to add?

Thank the participant for their time.
