# Supplementary material for: How Culture Enables Practice: Facilitators and Barriers to the Use of Total Intravenous Anesthesia in Pediatric Anesthesia
Source: Paediatr Anaesth. 2026 May 11;36(9):1078–88. doi: 10.1002/pan.70214 (PMC13460883; doi:10.1002/pan.70214)
Supplement: Supplementary file 2 [file PAN-36-1078-s002.pdf]

How culture enables practice – Facilitators and barriers to the use of total intravenous anesthesia in pediatric anesthesia

Appendix A – Survey consent and questions

Survey Consent

Study summary

Total intravenous anesthesia (TIVA) is the predominant mode of anesthesia in BC Children's Hospital (BCCH) operating rooms, with 57% of patients never being exposed to an inhaled anesthetic and an additional 20% of patients mainly having intravenous anesthesia. As such, BCCH has gained an international reputation for its training, use, and advocacy for this approach.

We aim to learn how departmental and organizational culture enables TIVA in children to inform implementation strategies and guide future teaching. For this, we are recruiting current and former anesthesiologists and anesthesia trainees at BCCH, who either had to adapt their practices to the local culture or who later tried to implement lessons learned at BCCH at another institution.

Your participation

Your participation in this survey study is voluntary. If you decide to participate, we will ask you to complete this survey, which includes a brief demographics section to tell us a little about yourself, such as your age, training, and past and current experience with TIVA.

Confidentiality

Your responses will be anonymous unless you disclose identifiable information in your free text answers. However, your responses will be de-identified at the close of the study using a unique code, and the project team will remove any information that could easily identify you. De-identified data will be shared with researchers worldwide, published, and used in presentations. The team may also use a quote from your comments.

Your confidentiality will be respected. However, research records identifying you may be inspected in the presence of the Investigator or designate and by the University of British Columbia / Children's and Women's Research Ethics Board so they can monitor the research. No information that discloses your identity will be published, removed, or released without your consent unless required by law. All study data will be stored within the BCCH Research Institute for five years after any publication of the survey results according to their approved security and privacy standards. Federal and provincial laws legally protect your privacy rights. You also have the right to access information about you and, if need be, correct errors in this information.

You can withdraw from the study anytime. However, we cannot remove your de-identified results if we cannot identify which response is yours or after your data are analyzed or shared with other researchers.

Participant remuneration

None will be provided.

Any questions?

If you have any questions about this study, you can contact the study's principal investigator, Dr Eleanor Reimer, at ereimer@cw.bc.ca. If you have any privacy concerns or questions about your rights as a research participant, don't hesitate to get in touch with the Research Participant Compliant Line in the UBC's Office of Research Ethics by e-mail at RSIL@ors.ubc.ca or by phone at 604-822-8598 (Toll-Free: 1-877-822-8598). Please reference the study number (H23-03017) when contacting the Complaint Line so the staff can better assist you.

We greatly appreciate your time in our attempt to learn more about the impact of institutional culture on implementing pediatric TIVA practices. This survey will take approximately 10-15 minutes to complete.

Eligibility question

1. Which of the following applies to you:
    - I am currently working or training at BC Children's Hospital
    - I have previously worked or trained at BC Children's Hospital
    - None of the above

If None of the above, "*Thank you for your time to consider participating in this survey. We are currently only surveying those with past or current experience at BC Children's Hospital*" and end the survey.

Survey questions

1. Your gender is
    - Male
    - Female
    - Agender
    - Two-spirited
    - Transgender
    - Gender Fluid
    - Non-Binary
    - Prefer not to answer
    - Other

2. How old are you?
    - < 30
    - 31 - 35
    - 36 - 40
    - 41 - 45
    - 46 - 50
    - 51 - 55
    - 56 - 60
    - 61 - 65
    - 66 –70
    - over 70

3. People come from different cultural and racial backgrounds. Please read the following categories and select all that apply to you:
    - Arab
    - Black (e.g., African, Haitian, Jamaican, Somali, etc…)
    - Chinese
    - Filipino
    - Japanese
    - Korean
    - Latin American
    - South Asian (e.g., Pakistani, Sri Lankan, etc…)
    - South East Asian (e.g., Vietnamese, Cambodian, Malaysian, Laotian, etc…)
    - West Asian (e.g., Iranian, Lebanese, Afghan, etc…)
    - White
    - Other
    - Prefer not to answer

    If other, please describe your background further __________

4. What is your current role as a physician?
    - Resident physician
    - Clinical fellow physician
    - Attending physician
    - Other, please specify in next question

    If other, please describe your current level of training __________

5. How many years have you been in practice?
    - 0-5
    - 5-10
    - 10-15
    - 15-20
    - 20+

6. In which setting do you typically practice?
    - Academic
    - Community

7. Approximately how many children do you provide perioperative anesthesia care for every year?
    - < 50
    - 50-100
    - 100-200
    - 200-300
    - 300-400
    - 400-500
    - >500

8. What was your preferred technique for <u>induction of anesthesia</u> in children <u>before</u> coming to BCCH? Please assign percentages out of 100 for each of these methods:
    - Intravenous induction with sedative pre-medication
    - Intravenous induction without sedative pre-medication
    - Inhalational induction with sedative pre-medication
    - Inhalational induction without sedative pre-medication

9. What was your preferred <u>maintenance</u> technique for children <u>before</u> coming to BCCH? Please assign percentages out of 100 for each of these methods:
    - Intravenous anesthesia
    - Inhalational anesthesia

10. What is your <u>currently preferred</u> technique for <u>induction of anesthesia</u> in children? Please assign percentages out of 100 for each of these methods:
    - Intravenous induction with sedative pre-medication
    - Intravenous induction without sedative pre-medication
    - Inhalational induction with sedative pre-medication
    - Inhalational induction without sedative pre-medication

11. What is your <u>currently preferred</u> maintenance technique for children? Please assign percentages out of 100 for each of these methods:
    - Intravenous anesthesia
    - Inhalational anesthesia

12. What are the most significant factors influencing your choice of anesthetic technique for pediatric patients?
    - Patient preference
    - Parental preference
    - Patient factors i.e., anticipated difficult airway, difficult IV access, etc.
    - Institutional culture or expectations
    - Availability of equipment, space, etc.
    - Pharmacology of anesthetic drugs
    - Other

    If other – please elaborate: ____________

13. Did your experience at BCCH contribute to your changes in pediatric induction or maintenance practices?
    - Yes
    - No

14. *If yes in #13,* What were the most compelling reasons to change*?*
    - Scientific evidence
    - Mentorship
    - Supportive learning environment
    - Departmental expectations
    - Positive experiences with this technique
    - Other

    If other – please elaborate: ____________

15. If no in #13, If not, what else changed your approach? ____________

16. What other factors might impact why anesthesiologists do or do not use TIVA more frequently?
    - Gaps in understanding the pharmacology of intravenous anesthetics
    - Lack of wide availability of equipment, such as depth of hypnosis monitors or (target-controlled) infusion pumps
    - Lack of support from an institutional perspective, such as parental presence in the operating room, expectations about using anxiolytic pre-medications, supportive peri-operative teams, etc.
    - Lack of space or time for IV placement before entering the operating room
    - Other

    If other – please elaborate: ____________

17. Which of the following factors do you consider critical to your success with awake IV cannula placement for intravenous induction of anesthesia in children?
    - Effective distraction
    - Preparing the family for IV induction
    - Parental presence
    - Support of the operating room team
    - Effective use of local analgesic cream
    - Adapting the approach to the individual child
    - The anesthesiologist's efficiency/speed of placing the IV
    - Other – i*f other, please describe*

18. What barriers do you encounter when attempting to place an awake IV for intravenous induction of anesthesia in children?
    - Needle phobia
    - Uncooperative child
    - Anxious parents
    - Ineffective use of analgesic cream
    - Unfavourable anatomy
    - Lack of support by the operating room team
    - Lack of support by the pre-operative team
    - Lack of parental presence at the induction location
    - Lack of time/resources for family education and preparation
    - Other – i*f other, please describe*

19. Assume you wanted to implement a TIVA program at your current or future institution. What would be the most significant barriers to success, and how would you suggest planning to overcome them?

20. Assume you wanted to implement a TIVA program at your current or future institution. What are the critical components in education, skills, and practices when bringing new learners or staff into your program, to enable them to successfully use TIVA in pediatric patients?

21. Do you have any additional comments about what determines your choice of anesthetic technique for induction or maintenance of anesthesia in children?

22. Do you have any additional comments about culture change in anesthesia (practice)?

23. Would you be interested in participating in an additional interview (over Zoom) to elaborate on your comments in this survey and share further insights with the study team?
    - Yes
    - No

24. If Yes to [23], Please enter your email address: _____________
    We will use email to communicate about this research study. The research team will use their best efforts to keep your information confidential. However, there are always some risks of disclosure when using email. You should be aware that some email services may store the contents of your email account outside of Canada, where privacy and data security standards may be different than in Canada. If you have questions or want to stop receiving research communication via email, please contact the study's principal investigator, Dr Eleanor Reimer, at ereimer@cw.bc.ca.
